# In vitro gastrointestinal gas monitoring with carbon nanotube sensors

**DOI:** 10.1038/s41598-023-50134-z

**Published:** 2024-01-08

**Authors:** Sahira Vasquez, Martina Aurora Costa Angeli, Andrea Polo, Alice Costantini, Mattia Petrelli, Enrico Avancini, Raffaella Di Cagno, Marco Gobbetti, Andrea Gaiardo, Matteo Valt, Paolo Lugli, Luisa Petti

**Affiliations:** 1 Sensing Technologies Laboratory (STL), Faculty of Engineering, Free University of Bozen-Bolzano, 39100 Bolzano, Italy; 2 Micro4Food Lab, Faculty of Agricultural, Food, and Environmental Sciences, Free University of Bozen-Bolzano, 39100 Bolzano, Italy; 3 Micro Nano Facility, Bruno Kessler Foundation, 38123 Trento, Italy

**Keywords:** Electrical and electronic engineering, Industrial microbiology, Nanobiotechnology

## Abstract

In vitro simulators of the human gastrointestinal (GI) tract are remarkable technological platforms for studying the impact of food on the gut microbiota, enabling continuous and real-time monitoring of key biomarkers. However, comprehensive real-time monitoring of gaseous biomarkers in these systems is required with a cost-effective approach, which has been challenging to perform experimentally to date. In this work, we demonstrate the integration and in-line use of carbon nanotube (CNT)-based chemiresitive gas sensors coated with a thin polydimethylsiloxane (PDMS) membrane for the continuous monitoring of gases within the Simulator of the Human Microbial Ecosystem (SHIME). The findings demonstrate the ability of the gas sensor to continuously monitor the different phases of gas production in this harsh, anaerobic, highly humid, and acidic environment for a long exposure time (16 h) without saturation. This establishes our sensor platform as an effective tool for real-time monitoring of gaseous biomarkers in in vitro systems like SHIME.

## Introduction

The gut microbiota is responsible for fermenting undigested carbohydrates, generating essential biomarkers within this process such as short-chain fatty acids (e.g., acetate, propionate, and butyrate) and gases (e.g., carbon dioxide ($CO_2$), hydrogen ($H_2$), methane ($CH_4$), ammonia ($NH_3$), and other volatile organic compounds (VOC). The presence of these biomarkers can be correlated with several nutritional and biological conditions. For example, $CH_4$ and $CO_2$ can be correlated with different lifestyles and feeding patterns of a given population^1^, while $NH_3$, commonly detected through exhaled breath, is potentially toxic to colon cells^2^ and has been correlated with halitosis^3^ and kidney failure^4^.

To investigate the gastrointestinal (GI) tract environment and its associated biomarkers, in vivo studies are crucial. However these studies often pose challenges, including ethical concerns, limited sample accessibility, and high costs (see Table S1). Additionally, they frequently yield non-uniform data due to the complexity and variability among individuals. Collecting intestinal biopsies is challenging, leading to the preference for fecal samples as the primary method of assessing the gut microbiota^9^. However, data from fecal samples have limitations in accurately representing the composition and activities of the gut microbiota in specific colon regions, failing to distinguish between luminal and mucosal aspects^9^. On the other hand, in vitro fermentation models offer a practical and promising technological platform for mimicking human microbial ecosystems. These models enable a continuous, real-time examination of how different diets affect the colon gut microbiota and its by-products^5–8^. Consequently, in vitro experiments using gastrointestinal simulators hold significant potential for eliminating interferences from factors like dietary habits and human physiology. This approach avoids the complexities and ethical considerations associated with human challenges, as claimed almost unanimously by the literature in the field^5–8,10^.

One of the most recognized and representative models is the Simulator of the Human Microbial Ecosystem (SHIME)^11,12^ from the company ProDigest (Gent, Belgium). It consists mainly of five interconnected reactors that simulate the human GI tract, starting from (1) the stomach, (2) the small intestine, and finally the colon with its three main sections: (3) ascending, (4) transverse, and (5) descending^7,8,13^. Versions of this system with increased complexity have also been developed, such as TWIN SHIME (two simultaneous experimental setups) and M-SHIME (including the mucosa microbiota)^14^.

To evaluate the composition and activity of the microbial community, as well as the conditions within the simulated GI tract during a SHIME experiment, common measurements include pH variation, nutrient absorption and transport, and the concentration of the metabolite (i.e. short-chain fatty acids) in the liquid medium present in the bioreactors. Despite the potential insights into the metabolic activities of gut microbes or the evaluation of the effects of dietary interventions, (e.g., prebiotics, or probiotics) that can be provided by analizing gas production during a SHIME experiment, few studies are reported in the literature. We believe that the limited research work done in this context is related to the lack of a simple-to-use and reliable system that allows the measurement of gases in a complex environment like the headspace of the SHIME bioreactors. Gases produced in such systems have previously been measured by using off-line analytical tools, such as gas chromatography coupled with mass spectrometry (GC-MS) or in-line instruments such as proton transfer reaction mass spectrometry (PTR-MS)^15^.

These analytical instruments are costly, bulky, and require complex sampling procedures and high-level operator skills,limiting their use. On the other hand, gas sensors are low-cost, easy to integrate in-line, and can work in real time. However, commercially available gas sensors, such as metal oxide sensors (MOS), and electrochemical sensors, are not conceived to operate in anaerobic environments, such as the SHIME^16^.

To the best of our knowledge, only two manuscripts report attempts to use commercially available gas sensors in a SHIME-like system, showing several limitations. A technical report by Langkvist et al.^17^ states that, in the attempt to use a MOS sensor to monitor gases in the SHIME, the sensor saturated immediately after a short test due to the anaerobic condition. On the other hand, optical sensors could potentially work in an anaerobic environment, however, they are typically more expensive and highly influenced by high humidity^15,18^.The difficulties mentioned in the above-cited manuscripts arise from the high humidity and anaerobic conditions in which SHIME operates. Nevertheless, these issues can be effectively addressed by employing sensitive materials capable of operating in oxygen-free environments, for instance, carbon nanotubes (CNTs).

CNT-based chemiresistive sensors have shown promise as candidates for realizing low-cost and low-energy demand gas sensors, as they can successfully work at ambient temperatures while showing high sensitivity towards different gas molecules^19–21^. The high surface-to-volume ratio, high mechanical strength, high response, lightweight, and low operating temperature make CNTs one of the most widely used functional materials for sensor fabrication^22,23^. While facing the challenge of low selectivity, as indicated by previous studies^24,25^, the literature has put forth various strategies. These encompass the introduction of metal nanoparticles decoration, the encapsulation of CNTs within polymer coatings, and covalent functionalization with diverse chemical groups, as exemplified by Schroeder et al., 2018^23^. Additionally, sensing in anaerobic conditions is not a limitation for CNTs, as their sensing mechanism is independent of the presence of oxygen molecules in the environment^26^.

In this work, we present a novel application of CNT-based chemiresistive sensors for monitoring total gas production within the SHIME in vitro system. CNT-based chemirisistive sensors were fabricated following our previous work^27^. Considering the distinctive characteristics of an in vitro system like SHIME, marked by high relative humidity (90–100$\%$) and the presence of numerous gases and biological media, we employed a thin PDMS membrane as a protective barrier for the sensor’s active layer, capitalizing on its hygroscopic and gas-permeable properties. The results of this pilot study showed the possibility of using the sensors for an extended exposure time (16 h) without degradation and saturation in a simple SHIME system composed of two bioreactors. Furthermore, the total gas output in the SHIME bioreactor measured with the developed sensor ranged from 49 to 300$\%$ after a 70 min exposure.

## Methods

### Sensor fabrication

#### Materials

To effectively detect gaseous biomarkers in an environment characterized by highly humid and acidic conditions and by a large presence of microbes, the materials should be carefully selected. A polyimide (PI) film, 50 μm thick (Kapton, from DuPont) was chosen as the substrate due to its chemical resistance, stability at high temperatures, low thermal expansion, and good mechanical properties^28^. Commercially available high-purity (90$\%$) single-walled carbon nanotubes (SWCNTs) (P3-SWNTs, Carbon Solutions Inc.) were used as active layers for their proven gas detection capability^23^. Silver (Ag) paste was used for the electrode deposition due to its electrical and physical properties (LOCTITE EDAG PF 410 EC), especially when used on flexible substrates. To allow permeation of gas molecules while preventing alteration of the sensing material from harsh environmental conditions and interference molecules, the sensing area and the bottom surface of the substrate were coated with a PDMS membrane (Sylgard R 184)^29–31^.

#### Fabrication process

**Figure 1:**
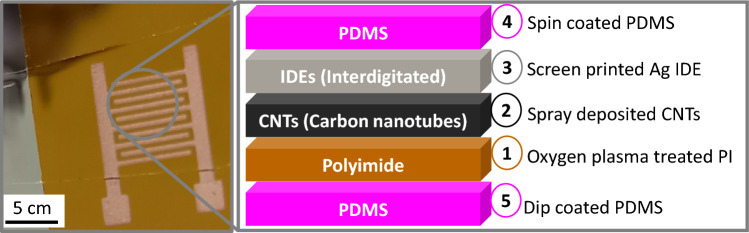
Image of the CNT-based chemiresitive gas sensor with its respective cross-section including the techniques used in each step of the fabrication process.

An image and a schematic illustration of the cross-section of the gas sensor realized are shown in Fig. 1. We prepared and deposited the CNTs using the methodology outlined in our prior work^27^. A thin film of SWCNTs was deposited on top of the oxygen plasma-treated PI by spray-coating a water suspension of SWCNTs with an air-assisted atomic spray nozzle (Krautzberger GmbH).

The Ag electrodes were then printed with an automatic screen-printing machine (C290Aurel automation s.p.a.), on top of twelve CNT layers, and these structures were sintered at 120$^{\circ }$C for 15 min in an oven. The selected design features two parallel, coplanar electrodes with a series of constant width fingers (300 μm) separated by a constant spacing (300 μm), and a specific length for each finger. Some sensors were coated with a thin layer of PDMS (PDMS coated CNTs) while others were left uncoated (Bare CNTs). For the preparation and deposition of PDMS, the two parts (base and cure) were mixed in a 10:1 ratio. The solution was then mixed manually and subsequently placed in a vacuum desiccator for degasification for 40 min. Three layers of PDMS were spin-coated (Quantum Design Srl) on top of the sensor at 130 rps/3s/20s and sintered in the oven at 100$^{\circ }$C for 30 min, and subsequently cooled at room temperature^29^. Finally, another PDMS layer was deposited beneath the PI (step 5 on Fig. 1) by dipping the sensor horizontally in the solution and then drying it as previously described. Beyond the PDMS membrane that covers the CNT layer, this extra layer beneath the substrate provides additional encapsulation for the entire sensor, creating a robust barrier from the external environment, which is also a distinctive feature as compared to our previous work and state of the art^27,29^.

**Figure 2:**
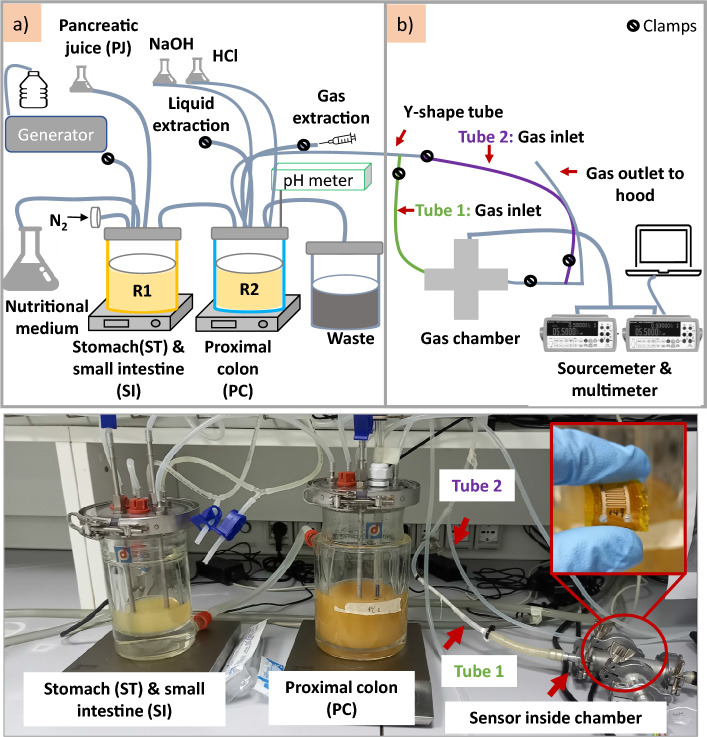
Schematic illustration of the SHIME experimental set up and its corresponding image; (**a**) model illustrating the first reactor (R1), representing the stomach (ST) and small intestine (SI) connected inline with the second reactor (R2) modelling the proximal colon (PC); (**b**) connections through a y shaped tube (tube 1 and tube 2) of the gas chamber. To test the sensor response and acquire the data, a source meter, a multimeter, a laptop, and a gas chamber were connected to the system.

### SHIME mounting set up

The SHIME model can be modified according to the specific digestive process of interest, thanks to the flexibility of its modular setup^14^. For our experiment, we developed a middle-term SHIME screening experiment composed of two bioreactors, the first reactor (R1) representing the stomach (ST) and the small intestine (SI), and the second reactor (R2) modelling the proximal colon (PC)^5^. To collect the waste, a container connected to the R2 was located beneath the supporting table. An image of the system while performing the experiments and its corresponding schematics is illustrated in Fig. 2. The experiments were carried out for a total of 13 days, starting with the inoculation of the bacteria in R2 (Day 0) until Day 13.

Following the procedure described by Molly et al, to initiate the growth of the microbial ecosystem, on Day 0, 5g of the fecal sample was mixed with a buffer to prepare a homogeneous suspension that was used as inoculum^32^. A sterile nutritional medium (SHIME R growth medium PD-NM002B, Prodigest) with a pH of 2 and stored at 4$^{\circ }$C was pumped three times a day as a food source for the microbial community. Pancreatic juice (PJ) was prepared with oxygall (Bile Bovine, Sigma Aldrich, SLCG9142), sodium hydrogen carbonate ($NaHCO_3$) (Supelco, Germany) and pancreatin (Sigma Aldrich, P3292-100G). Peristaltic pumps were used to transfer the lumen from R1 to R2 and subsequently to the waste bottle.

One of the great advantages of the SHIME model used is that it provides a highly standardized and controlled environment. The system automatically controls important parameters, including temperature, pH, the flow of the nutritional medium, PJ, and anaerobic conditions, ensuring the survival of the microbial community.

Specifically, the system ensures a constant temperature of 37 $^{\circ }$C throughout the entire experiment. The pH of R2 was set between 5.6 and 5.9. This range was automatically adjusted by adding either NaOH or HCl and constantly monitored with an integrated pH meter. 5 ml of the liquid media were extracted every afternoon to compare and validate the pH reading on the monitor with an external pH meter. The anaerobic condition of the reactors was ensured by flushing oxygen-free nitrogen gas in two ways: manually by pressing the nitrogen button in the program (after introducing a sensor or opening the chamber) or automatically every day at 7:00 am (see the program in Table 1). Furthermore, a gas leakage test was performed every day by folding all connected tubes while flushing constant nitrogen and checking the pressure.

Table 1 illustrates the activities of a SHIME complete cycle divided into three columns. The first and second columns indicate the starting and ending time of each action, while the third column describes the action itself. The first activity started with the control of pH and temperature, followed by the three feeding cycles and their respective actions.

**Table 1:** Activities of one daily SHIME cycle copied and modified from the software of the model.

Start time	End time	Action
00:00:00	23:59:59	Fixed pH controller Action (PC - 5.60/5.90)
00:00:00	23:59:59	Temperature Alarm (Temperature Sensor 1)
**01:00:00**	**01:30:00**	** Pump Action (Feed - 4.67 ml/min):** 1st **meal**
02:30:00	02:45:00	Pump Action (Pj- 4.00 ml/min)
04:00:00	05:15:00	Pump Action (st/pc- 3.50 ml/min)
04:00:00	05:35:00	Pump Action (pc/w- 3.50 ml/min)
07:05:00	07:05:10	Flow Verification Action (Min Flow: 1.00 l/min)
07:00:00	07:10:00	Flush Nitrogen Action (SHIME 1)
**09:00:00**	**09:30:00**	**Pump Action (Feed - 4.67 ml/min):** 2nd **meal**
10:30:00	10:45:00	Pump Action (Pj- 4.00 ml/min)
12:00:00	13:15:00	Pump Action (st/pc- 3.50 ml/min)
12:00:00	13:35:00	Pump Action (pc/w- 3.50 ml/min)
**17:00:00**	**17:30:00**	**Pump Action (Feed - 4.67 ml/min):** 3rd **meal**
18:30:00	18:45:00	Pump Action (Pj- 4.00 ml/min)
20:00:00	21:15:00	Pump Action (st/pc- 3.50 ml/min)
20:00:00	21:35:00	Pump Action (pc/w- 3.50 ml/min)

This cycle was automatically controlled by the system and could be easily monitored in the program to verify the action that was taking place at a specific time during the day. The first two actions, which were related to pH and temperature checks, were performed continuously for 24 h. Temperature was verified with a temperature sensor that was located near the water bath pipe. In total, 140 ml of nutritional medium and 60 ml of PJ were fed three times a day. Each feeding cycle (1st meal, 2nd meal, and 3rd meal) started with the transfer of the nutritional medium located in a small fridge (4.6 ml/min) to R1. Then PJ (4 ml/min) was added to R1. After that, the content of R1 was transferred to R2 (3.5 ml/min) and subsequently to the waste bottle (3.5 ml/min).

### Gas sensing set up and protocols

Unlike testing a gas sensor under controlled laboratory conditions, where the species and concentration of the gas are previously known and constant over exposure time, in the SHIME the gas production is naturally happening due to the undergoing microbial activity. Therefore, to systematically evaluate the sensor response, a SHIME-specific protocol was developed, with details provided below.

To understand the impact of the presence of the PDMS membrane on the sensor response, we tested both devices covered with the PDMS membrane and uncovered devices. Both sensors were inserted into the chamber on day 1, after inoculation. The time window for the different tests was selected based on the ongoing actions in the system (see Table 1). Two slots were selected, the first one after the 2nd meal from 13:35 to 17:00 (short test) and from the 3rd meal of one day starting at 17:00 to the 1st meal of the next day at 9:00 (overnight test). To evaluate the sensor response in the SHIME two different tests were performed:

- **Active sensing**: In this condition, the gases accumulated in R2 were brought in contact with the sensor in the gas chamber (clamps of tube 1 were opened and clamps of tube 2 were closed) by flushing nitrogen ($N_2$) gas (2000 ml/min) for 5 min. $N_2$ was used as a carrier to increase the gas flow speed. This test was meant to mimic the tests typically done in a controlled environment (with known gas concentrations),
- **Passive sensing**: In this condition, the gases accumulated in R2 naturally diffused inside the gas chamber for ca. 16 h without nitrogen flush, except for the daily automatic flush scheduled at 7:00 am.

The recovery time of a gas sensor is desired to be as fast as possible to enable quick recovery to the baseline condition. For CNT-based gas sensors, a common method to achieve a fast recovery is to heat the sensing layer, in a process known as active recovery, which induces a faster release of the gas molecules adsorbed on the surface of the film^33^. However, this method increases power consumption. To study the capability of the developed sensor to operate also without active recovery and its saturation point, the devices were tested in both conditions: (1) with active recovery and (2) with passive recovery (no heating). For the active recovery, the sensor was reset to its baseline resistance by increasing the temperature to 60 $^{\circ }$C by using a Peltier element, while in the passive recovery, the sensor behavior was evaluated by keeping the temperature always at 25 $^{\circ }$C.

Chemiresistive gas sensors are a class of chemical sensors that change their resistance in the presence of certain types of gases. In this work, the sensor response is presented as the absolute sensor resistance (R, [K$\Omega$]) and the normalized resistance (NR, [au]). The NR was calculated with Eq. (1).

1 $$\begin{aligned} NR=\frac{R_f-R_i}{R_i} \end{aligned}$$

where $R_i$ is the measured initial resistance of the sensor before the active sensing (for the short test) or after 10 min of measurement (for the long test), while $R_f$ is the measured resistance at the end of the active sensing.

Within a SHIME experimental session, conditions in the system are known to evolve following the expected changes of the biological environment, thus results of the sensor measurement are presented on individual days, instead of day average. Qualitative and quantitative comparisons are made only between similar sensors and tests.

### Data acquisition

The elements for the acquisition of data are shown in the schema of Fig. 2b. The sensor resistance reading was acquired with a digital multimeter (Keithley DAQ6510). The heating system was powered using a sourcemeter (Keithley 2602B). Additionally, a laptop and a gas chamber were connected in line with the SHIME setup. Sensors inside the gas chamber were placed in a module including a Peltier element and a Pt100 to control the temperature during the test. The gas chamber was connected to the SHIME setup via a y-shaped tube where the gas from the reactor crossed and passed through the sensor, as shown in Fig. 2. Clamps were located in both tubes either to allow the gas to flow through the gas chamber or to deviate the gases directly to the fume hood. To preserve the anaerobic conditions of the system, before introducing a sensor into the gas chamber, the clamps of tube 1 were closed, and those of tube 2 were opened.

### Data analysis

Sensors were evaluated based on their response to the corresponding increase or decrease in gas production induced by microbial activity. Specifically, the sensor response to the activities such as feeding (1st meal, 2nd meal, and 3rd meal), when an increase in gas production is expected, was analyzed. These activities (Table 1) were used as a reference to overlap the sensor response with the ongoing events. It is worth noting that since the SHIME system inherently exhibits dynamic behavior, where gas production is continually influenced by the ever-evolving microbial community, each day represents a distinct condition. Thus, we decided to report the single test data day by day because, in this specific context, we acknowledge the inherent limitation of comparing tests performed on different, even if consecutive, days.

### Gas analysis by mass spectrometry

To investigate the composition of the gas mixture emitted by the SHIME, a sample of the SHIME’s atmosphere was collected by using a gas-tight syringe (see Fig. 2a) and a plastic sample bag and subsequently analyzed using quadrupole mass spectrometry and proton-transfer-reaction time-of-flight mass spectrometry (PTR-TOF-MS).

Quadrupole mass spectrometry measurements were performed with a Pfeiffer Vacuum QMS 200 equipped with a tungsten grid ion source. A custom-made differential pumping system allows constant fluxing of gas inside the gas phase analysis chamber with an Alicat MC-series mass flow controller through a capillary stainless steel tube (120 μm internal diameter). The background spectrum was acquired with a constant flow of 2 sccm of $N_2$, then the sample was injected into the system using a suitable syringe at the same flow rate. The gas phase analysis chamber was heated at 100 $^{\circ }$C to avoid any vapor condensation.

Furthermore, the sample was analyzed using a PTR-TOFMS instrument (Kore Technology) equipped with a Hollow Cathode Glow Discharge Ion Source (GD), which could be started with water for the proton reaction or with other gas (Ar, Kr) for the charge transfer reaction^34^. In this experiment, $H_3O^+$ was used as an ion source. The pressure in the reaction chamber was approximately 0.5 mbar and the inlet flow was set to 4 sccm, using an ALICAT mass flow controller. Room air filtered with activated carbon was injected into the PTR-TOF-MS and analyzed as a reference.

## Results and discussion

### Sensors’ response: short test

**Figure 3:**
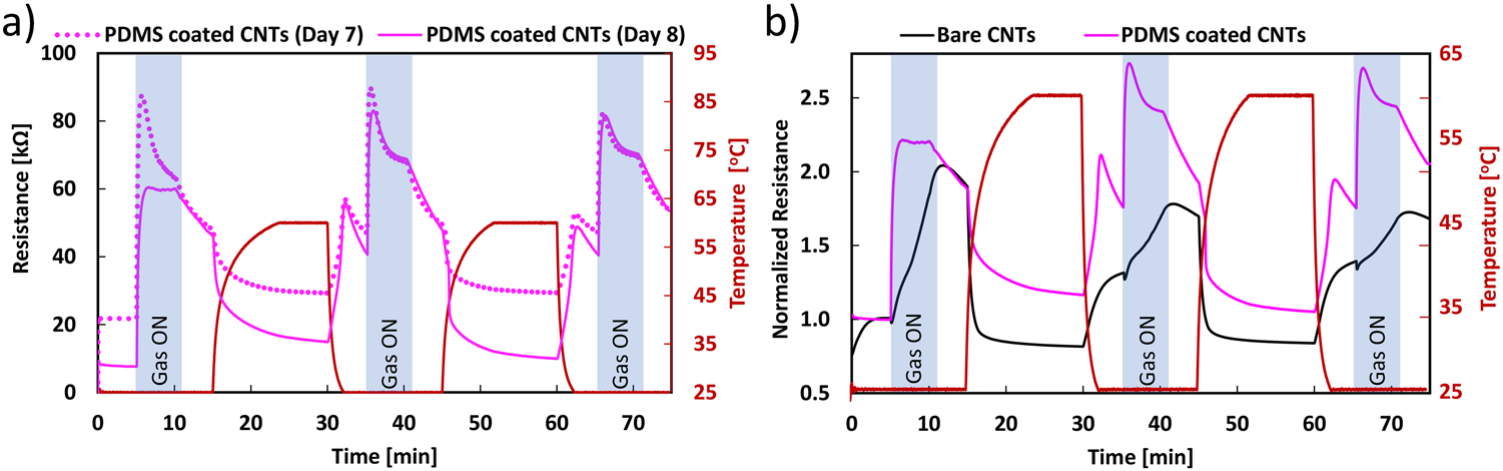
Sensor response during a short test: (**a**) resistance vs. time while performing active sensing and recovery of the PDMS coated CNTs sensor on day 7 and day 8, (**b**) comparative normalized resistance vs. time for a bare CNTs (day 6) and the PDMS coated CNTs sensor (day 8) during active sensing and recovery.

Fig. 3a illustrates the temporal response of two CNT sensors, each coated with PDMS membranes of 12 μm thickness, tested on separate days. Both sensors were subjected to active sensing and active recovery conditions. In the initial 15 min of each test, the temperature was set to 25$^{\circ }$C: 5 min with the gas OFF, followed by 5 min with the gas ON, and finally 5 min with the gas OFF. Then, in the following 15 min, the temperature was increased to 60$^{\circ }$C to induce recovery. This cycle was repeated twice more, from 30 to 45 min and from 60 to 75 min. Notably, during in each gas exposure phase (referred to as E1, E2, and E3 when the gas was ON), the sensors exhibited a rapid response upon contact with the gaseous biomarkers emitted by R2.

We conducted this test to assess sensor responses under conditions similar to our prior experiments in a controlled gas chamber^27^ and to evaluate the membrane’s impact in the SHIME environment. The sensors exhibited responses to gas exposure similar to what was previously reported. This indicates the ability of CNTs to function as a fast-responding gas-sensing material, even under anaerobic conditions and in complex environments such as SHIME. Furthermore, after each exposure, thermal recovery returned the sensor to its initial condition. In the SHIME test, the response displayed peak values approximately within the initial 1 to 2 min, gradually decreasing before the gas flow was turned OFF. This is likely due to a gradual decrease in the concentration of different response-inducing gasses in the $N_2$ carrier. Of interest is the observation that the sensors responded similarly even when tested on two different days (days 7 and 8).

Figure 3b shows the response to the total gas produced in the SHIME reactor, measured using both a bare CNTs sensor and a PDMS coated sensor on days 7 and 8 of the experiments. The graph reveals two primary differences in sensor response. Firstly, the coated sensor exhibits quicker and higher resistance upon gas contact, whereas the uncoated CNT sensor responds more slowly. A quantitative comparison of the response to total gas production measured with two bare CNTs and two PDMS coated CNTs sensors on four different days is presented in Table 2. Understanding total gas production in the bioreactors is vital for optimizing, controlling, and monitoring the bacterial fermentation bioprocess^35^. This data provides insights into the kinetics of fermentation, allowing for real-time adjustments to enhance efficiency and yield in the system^36^. Real-time and inline monitoring of total gas production in the SHIME serves as a valuable indicator for estimating product yield, troubleshooting irregularities, and maintaining quality control on each experiment. Additionally, knowledge of total gas production is essential for researchers and engineers working on fermentation on going in in vitro systems, offering a comprehensive understanding of the process dynamics and aiding in assessing the impact that diets have on the microbial community^37^. As previously clarified, we have refrained from presenting average values because the sensor measurements correspond to different days, and the total gas production reflects momentary microbiological activity. Nonetheless, it is noteworthy that during the days we measured with the PDMS-coated sensor, responses fell within a range of 49–300$\%$. In contrast, when employing CNT sensors, we observed lower values starting at 13$\%$, with the highest recorded value being 103$\%$.

Huang and colleagues achieved analogous findings in their research, employing a commercial MOX sensor to detect volatile organic compounds (VOCs). Their approach involved encapsulating the sensor with polycaprolactone and parylene C to facilitate VOC testing within a GI tract model^38^. Their investigations yielded noteworthy outcomes, as they observed that the encapsulation of the gas sensor with Parylene C, specifically using thicknesses of 10 μm and 20 μm, led to a significant reduction in sensor noise. Furthermore, this encapsulation technique concurrently elevated the precision of detection. In essence, their research underscores the beneficial impact of such encapsulation strategies in enhancing sensor performance and accuracy.

PDMS has a similar effect as Parylene C on top of the CNTs. In fact, as shown in a previous work^29^ the presence of a PDMS membrane over the CNT network demonstrated a favorable effect on sensor response characteristics, in terms of sensitivity and selectivity. This phenomenon can be attributed to the dual attributes of the PDMS membrane: its gas size-dependent permeable nature and the inherent hydrophobic property^39,40^. Thus, the membrane enhances the sensor performance by simultaneously engaging the gas interaction and protecting the active material against environmental factors, including external perturbations and contaminants, thereby preserving the integrity of the CNT network. Certainly, a comprehensive examination of the membrane’s susceptibility to various external factors, including contaminants, perturbations, and water vapor, warrants further investigation.

**Table 2:** Normalized gas response of two PDMS coated and two Bare CNTs sensors during three exposure cycles to the total gas production of the SHIME reactor.

Gas	PDMS	PDMS	Bare CNTs	Bare CNTs
Exposure	(NR/%)	(NR/%)	(NR/%)	(NR/%)
E1	3.00/300.05	1.21/120.59	1.03/103.30	0.93/93.10
E2	0.89/89.37	0.55/55.30	0.38/37.68	0.14/13.83
E3	0.72/72.26	0.49/49.14	0.28/27.75	0.13/13.36

It is worth noticing that in Fig. 3a, b, the use of temperature during the active recovery plays a major role in the resistance value. A representative temperature response of the PDMS coated CNTs sensor at the corresponding temperatures used during the test, i.e. 25$^{\circ }$C y and 60$^{\circ }$C is illustrated in Fig. S1. Generally, an increase in temperature induces a decrease in resistance and vice versa. For instance, the sensor starts with an initial resistance of 33 K$\Omega$ at 25$^{\circ }$C that then drops to approximately 25 K$\Omega$ when the temperature rises to 60$^{\circ }$C. The flat and constant resistance of the sensor at 25$^{\circ }$C (from 30 to 50 min) should be noticed on the graph, as this demonstrates that the response to gases (shown in the figures below) can be easily distinguished from temperature since it is faster and more pronounced.

### Sensors’ response: overnight test

**Figure 4:**
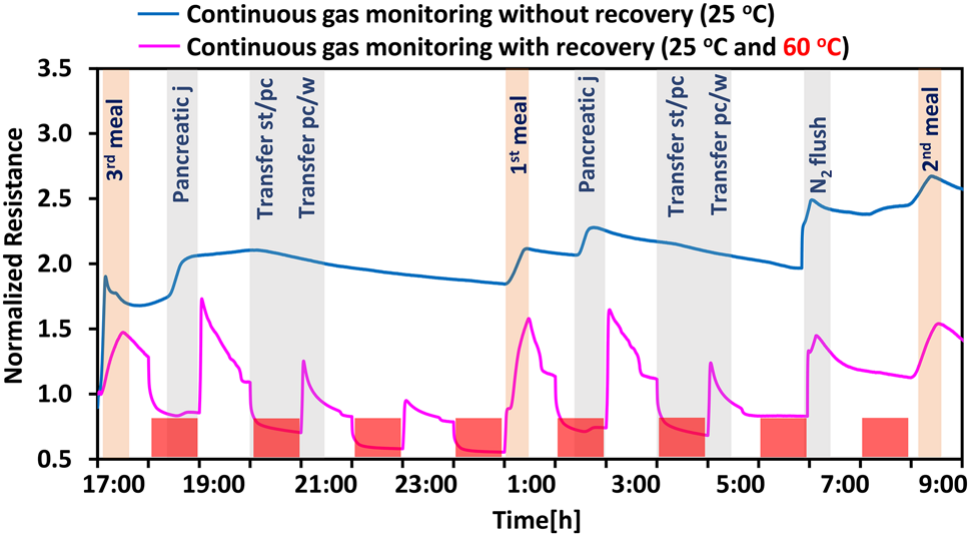
Normalized response versus time during passive sensing with recovery and without recovery of two PDMS coated CNTs sensors.

Throughout the day, bacteria receive fresh nutrients every 3 hours after the start of each cycle. Consequently, they exhibit increase activity during these hours (4-7, 12-15, and 20-23) compared to time slots when the PJ pump is inactive and microbes are less active (e.g. between 22-1, 7-9, and 15-17). The microbial community is particularly active at three specific moments during the day when they start fermenting the freshly supplemented nutritional medium (at 17:15, 01:15, and 9:15). This heightened activity leads to an expectation of increased gas production in these time slots. Figure 4 shows the continuous gas monitoring by the PDMS coated CNT sensor during a complete SHIME cycle on day 10, performing the active recovery at constant intervals (every 60 min), and on day 11 with passive recovery (no heating). The test started at 17:00 with the 3rd meal and ended the following day after the 2nd meal at 9:00. The temperature alternated between 25$^{\circ }$C and 60$^{\circ }$C in a 2 h cycle during the test with recovery and remained constant at 25$^{\circ }$C for the test without recovery.

As illustrated in the graph, during continuous gas monitoring with recovery (pink line) test, certain SHIME activities, specifically the transfer of content from R1 to R2 after the 3rd meal, and the timeline after the transfer of content from R2 to waste, overlapped with the active recovery time at 60$^{\circ }$C, resulting in being hidden by the resistance change due to the temperature. Despite this overlap, the curve clearly shows a response of the sensors to the different activities of the microbial community. This is more evident in the sensor tested without active recovery (blue line). In fact, it is visible that during the three feeding times (at 17:15, 01:15, and 21:15), and during the PJ addition (18:00, and 2:00), the sensor response is higher compared to the rest of the activities. Meanwhile, the resistance slowly decreases during the expected inactivity time of the bacteria, specifically between 20:00 and 1:00, and 3:00–7:00. It is also worth noticing that the sensor tested without active recovery did not show any saturation and presented a small drift of circa 0.002$\%$ (calculated as the difference between the resistance value at 23:00 (5h after the 3rd meal) and at 7:00 (5h after the 1st meal) over a 16 h period. The graph indicates minimal microbial activity from 3:00 am to 6:59 am, but the introduction of $N_2$ flow is significant at 7:00am. Here, the increase in resistance is pronounced, probably due to the transport of gases accumulated in the bioreactors during the previous time, suggesting the presence of various gas byproducts from microbial activity. Additionally, compared with the previous two meals, here we can observe a higher sensor drift (0.2$\%$), probably due to the very long exposure time.

### SHIME system evaluation

#### Functionality

Integrating additional devices within the SHIME may impact the system, necessitating slight modifications to the architecture of the original setup to accommodate the sensing devices under test. Consequently, certain parameters such as temperature, pH, nutritional medium, PJ, and anaerobic conditions, indicative of the microbial community´s survival, were evaluated daily and correlated with the sensing activities performed. Regarding the evolution of temperature and pH, values remained constant within the expected range throughout the experiment. The consumption of nutritional medium and PJ also aligned with anticipated values. Specifically, from the 1st meal of day 1 to the 3rd meal of day 7, a total of 2800 ml and 1200 ml of nutritional medium and PJ were consumed, respectively, aligning with the standard functioning of the peristaltic pumps and the expected feed delivery and consumption in the system. The acid/base maintained the pH within the correct range, with a total volume of 38 ml and 26 ml of acid and base consumed from day 1 to day 7, respectively. Unlike the fixed values of pH and PJ, daily acid and base consumption vary.

#### Gas analysis

**Figure 5:**
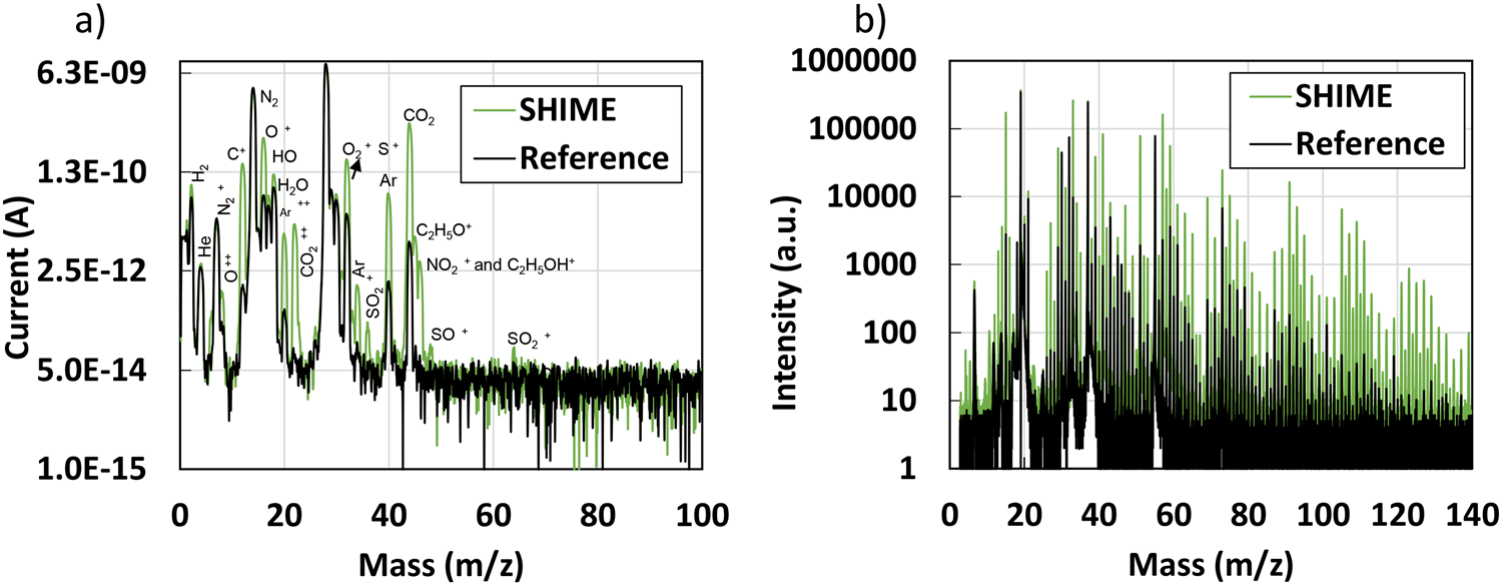
Spectra of the reference and SHIME sample obtained from the (**a**) Quadrupole MS analysis and (**b**) PTR-TOFMS analysis.

Despite the affinity and higher response of pristine CNTs to certain gases (e.g., $NH_3$) compared to others (e.g., $CH_4$, $CO_2$)^23,27^, in this study, we have chosen to define the sensor response as the “total gas response” rather than specifying individual gases. This decision is motivated by the complex and diverse array of gas species present within the system, making it more suitable to provide a comprehensive overview of the sensor’s performance rather than focusing on individual gases. The gas profile of the SHIME system was quite diverse, with more than 20 different gas molecules identified. Figure 5a shows the gas profile of the SHIME sample measured through Quadrupole MS. Several peaks emerge from the graph compared to the background sample ($N_2$). In particular, water vapor (8,16,17,18 m/z), $CO_2$ (6,12,28,29 m/z), NO (14,16,30 m/z), $NO_2$ (14,16,46 m/z), $SO_2$ (32,48,64 m/z), $H_2S$ (32,33,34 m/z) and several VOCs. The peaks for  $H_2$ (1,2 m/z) and argon (20,36,38,40 m/z) are probably due to the presence of ambient air in the sample bag. Gases are experiment-specific, meaning that the gas species and concentrations reported in a specific scientific work are related to the specific food, treatment, or faecal donor used in the study. In terms of typical gas output, a prior study of a complete SHIME experiment (6 reactors) revealed a total gas production of 2739.66 ml/day, with 18.32 mmol/day of $CO_2$ and 2.0 mmol/day of $CH_4$^1^. Even under different conditions, this investigation revealed that total gas concentrations in the SHIME might be extremely high, which could explain the relatively high response that our sensors acquired in this work (ranging from 49.14 to 300$\%$).

PTR-TOF-MS provides complementary information compared to Quadrupole MS analysis, owing to the low fragmentation rate of the analyzed gases. Figure 5b displays the spectra of the reference sample and the SHIME sample in the 0-140 m/z range. Notably, the primary differences between the two spectra are observable at m/z values higher than 80, attributed to the presence of fatty acids in the SHIME sample. It is also important to mention that both samples exhibit a high concentration of species derived from the use of $H_3O^{+}$ as a source of protons (positive charge). Specifically, there is a saturation of the signal at m/z=19 ($H_3O^{+}$), alone with robust peaks at m/z 18, 20, and 21 (see Fig. S1a). Furthermore, due to the clusterization of $H_2O$ molecules (the precursor of $H_3O^{+}$ in the PTR-TOFMS) with $H_3O^{+}$, strong peaks are present in both spectra at m/z 37 ($H_2O$-$H_3O^{+}$), 55 ($H_2O$-$H_2O$-$H_3O^{+}$), 73 ($H_2O$-$H_2O$-$H_2O$-$H_3O^{+}$), 91 ($H_2O$-$H_2O$-$H_2O$-$H_2O$-$H_3O^{+}$). Therefore, for the purpose of the spectra comparison, the peaks at these m/z values (and related peaks due to oxygen isotopes) are not considered.

**Figure 6:**
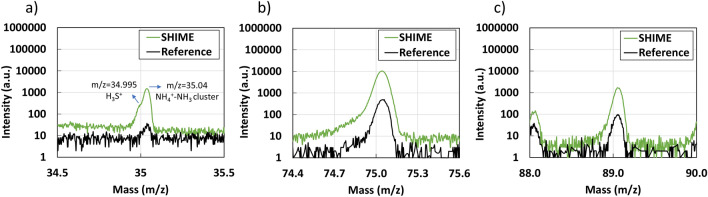
Highlited peaks from the PTR-TOF-MS spectra: (**a**) sulfonium ($H_3S^+$ m/z = 34.99) and $NH_4^+-NH_3$ (m/z = 35.04), (**b**) propionic acid ($C_3H_6O_2H^{+}$, m/z = 75) and (**c**) butanoic acid ($C_4H_8O_2H^{+}$, m/z = 89).

Figure 6a shows the appearance of the $H_3S^+$ peak (m/z = 34.99), attributed to the presence of hydrogen sulfide ($H_2S$) in the SHIME sample. In both the reference (weak) and SHIME sample (strong) a peak is observed at m/z = 35.04, probably due to the presence of $NH_4^+-NH_3$ clusters^41^. It's noteworthy that this peak has been recognized only in a couple of previous works, and its origin has not yet been fully confirmed. Furthermore, the identification of significant carboxylic acids produced in the colon through bacterial fermentation of dietary fibers is evident in the SHIME sample. This includes propionic, butanoic, hexanoic, and heptanoic acids. The peaks corresponding to propionic and butanoic acids are illustrated in Fig. 6b,c respectively. Further analysis, along with figures illustrating other important compounds found in the sample is presented in Supplementary Information.

## Conclusion

In this article, for the first time, carbon nanotube-based chemiresitive gas sensors were integrated into the highly standardized in vitro model, SHIME. The SHIME system is widely used to study the impact of the food diet on the gut microbiota. Here, the dynamic response of the gas sensor uncoated and coated with a PDMS membrane during multiple days of SHIME running experiments is presented. The main findings demonstrate that CNTs allow continuous monitoring of the different phases of gas production in this harsh, anaerobic, highly humid, and acidic environment, for a long exposure time (16 h), without saturation and significant drift. This is a great advantage and improvement in the standardization and control of the system, as well as demonstrating CNTs as a suitable material to sense under fully anaerobic conditions. Furthermore, our findings also showed that in a complex environment, the PDMS coated sensor showed a higher response compared to the bare CNT sensor, as we demonstrated before with the controller test^29^, experimentally demonstrating that the membrane has the function to protect the sensing layer from the surrounding harsh environmental conditions. In conclusion, in this preliminary research work, we demonstrate the possibility to monitor inline and in real-time the undergoing gaseous biomarkers produced by the microbial community of the GI tract model. Future improvements to this study include the need to improve sensor selectivity as well as the integration of all electronics and electrodes into one probe unit. Additionally, comprehensive sensor testing under various conditions, including but not limited to scenarios involving bending or other physical stresses, is necessary to ensure its durability and versatility.The achievement of this preliminary study encourages further research and development toward monitoring inline and real-time through sensor technologies all relevant biomarkers originating through microbial fermentation. First, this will eliminate the need to wait days to make decisions about the status of the experiments when using analytical techniques, and second, it will enrich our knowledge of the interesting food-gut axis.

### Supplementary Information

Supplementary Information.

## Data Availability

All data generated or analysed during this study are included in this published article and its supplementary information files.
