# Supplementary material for: In vitro gastrointestinal gas monitoring with carbon nanotube sensors
Source: Sci Rep. 2024 Jan 8;14:825. doi: 10.1038/s41598-023-50134-z (PMC10774382; doi:10.1038/s41598-023-50134-z)
Supplement: Supplementary file 1 — Supplementary Information. [file 41598_2023_50134_MOESM1_ESM.pdf]

In vitro Gastrointestinal Gas Monitoring with Carbon Nanotube Sensors

Sahira Vasquez[1,*], Martina Aurora Costa Angeli[1], Andrea Polo[2], Alice Costantini[2], Mattia Petrelli[1], Enrico Avancini[1], Raffaella Di Cagno[2], Marco Gobbetti[2], Andrea Gaiardo[3], Matteo Valt[3], Paolo Lugli[1], and Luisa Petti[1,*]

[1]Sensing Technologies Laboratory (STL), Faculty of Engineering, Free University of Bozen-Bolzano, 39100, Bolzano, Italy
[2]Micro4Food Lab, Faculty of Agricultural, Food, and Environmental Sciences, Free University of Bozen-Bolzano, 39100, Bolzano, Italy
[3]Micro Nano Facility, Bruno Kessler Foundation, 38123 Trento, Italy
[*]Corresponding author. E-mail:svasquezbaez@unibz.it, luisa.petti@unibz.it

SUPPLEMENTARY INFORMATION

Table S1. In vivo vs invitro approach

Limitations of in vivo trials
Inter-individual differences based on dietary habits
Fecal samples different from gut microbiota
Difficulty to map all dietary components
No gut microbiota differentiation between lumen and mucosa
Problems to extrapolate data only from bread intake
Poor information on the gut microbiota from colon tracts without using biopsies
SHIME capability
Unique multi-compartmental dynamic model of the GIT
Differentiation of colon tracts
Fecal inoculation from only one donor (high reproducibility)
In vitro reproduction of mucosal environment
Adaptation of fecal microbiome to the colonic conditions
Possibility to simulate different target groups
Interferences from other dietary factors are excluded
Human physiology overlaps attenuated
Possibility of long-term experiments

S1 Sensor supplementary data

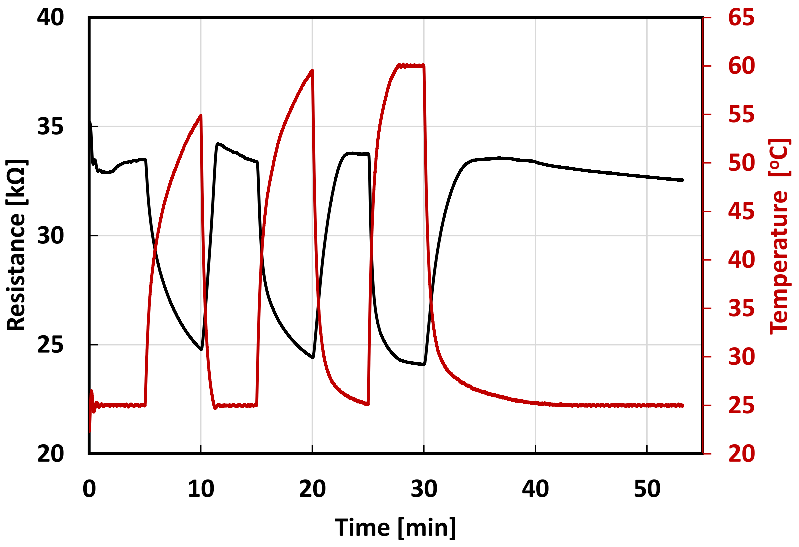

Figure S1. Resistance vs time at five minutes alternating temperature cycle of 25°C and 60°C, respectively. This cycle was performed on all sensors every day prior to the exposure to the gases.

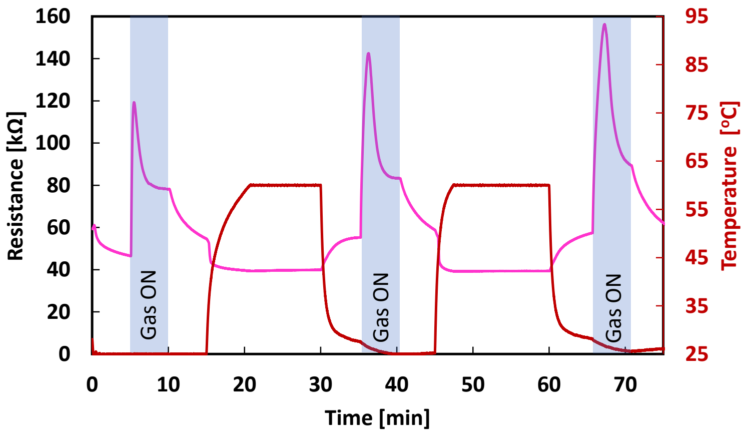

Figure S2. Short test: resistance vs. time when performing active sensing and recovery of the PDMS coated CNTs sensor at day 4.

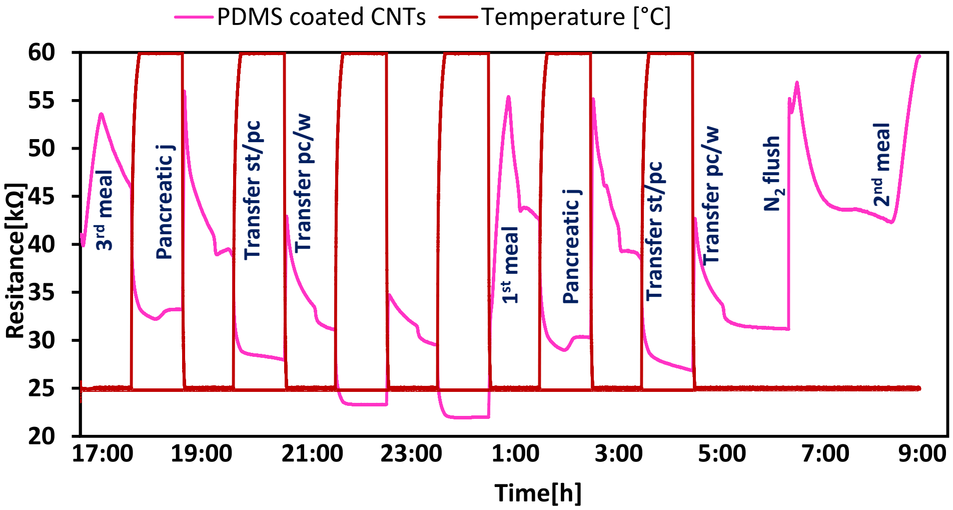

Figure S3. Resistance vs. time when performing passive sensing with recovery of one PDMS coated CNTs sensor at day 6.

S2 Gas analysis supplementary data

Though a very low molecule fragmentation is expected during the protonation and subsequent acceleration of the molecules in the PTR-TOFMS, a high concentration of VOCs can lead to the appearance of $C_2H_2^+$ (m/z = 26), $C_2H_3^+$ (m/z = 27), $C_2H_4^+$ (m/z = 28) and $C_2H_5^+$ (m/z = 29) fragments (Fig. S4b). Furthermore, the peak at 29 m/z is also probably indicative of the presence of ethylene (C_2H_4) in the SHIME sample, which will be protonated to $C_2H_5^+$. Fig. S4c shows the peaks at m/z = 30. As can be seen, in both the SHIME sample and reference there is the presence of NO^+ peak at m/z = 30.006. Furthermore, in the SHIME sample, a peak appears at m / z = 30.34, probably due to the presence of methylene imine (CH_3NH^+). In the SHIME sample, peaks related to formaldehyde (CH_2OH^+, m/z = 31) and methanol ($CH_3OH_2^+$, m/z = 33) are clearly evident (Fig. S4d). On the contrary, the peak related to O_2 (O_2^+, m/z = 32) is more prominent in the reference. The low intensity of the O_2^+ peak suggests that it probably belongs to the ion source (H_2O/H_3O+) instead of being present in the SHIME sample. Fig. S5a highlights the presence of both protonated formic acid ($CH_2O_2H^+$, m/z = 47.014) and protonated ethanol ($C_2H_6OH^+$, m/z = 47.049) in the SHIME sample. On the other hand, Fig. S5b shows the presence of both propylamine ($C_3H_9NH^+$, m/z = 60) and acetic acid ($C_2H_4O_2H^+$, m/z = 61) in the SHIME sample, but with an intensity of the peaks that are comparable to the reference. An intense peak related to dimethyl sulfide ($C_2H_6SH^+$) has been also identified in the SHIME sample (Fig. S5c). in addition to formic and acetic acids, other carboxylic acids have been identified in the SHIME sample, pentanoic acid (Fig. S5d), hexanoic acid (Fig. S6a) and heptanoic (Fig. S6b). Other VOCs are well identified in the SHIME sample with the PTR-TOFMS analysis, including terpenes (e.g. isoprene and pinene/limonene, Fig. S6c,d) and benzene derivatives (e.g.

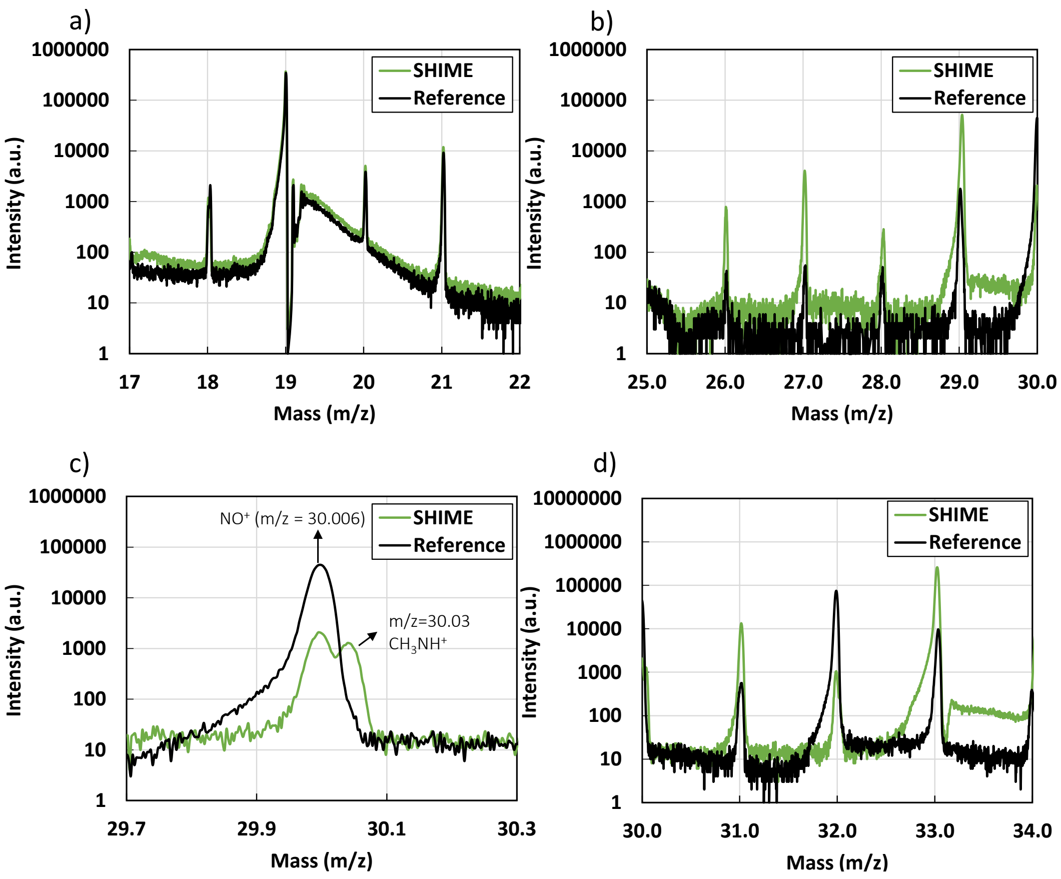

Figure S4. (a) H_3O^+ (m/z = 19) and related satellite peaks (18, 20, and 21), (b) VOCs fragments: $C_2H_2^+$ (m/z = 26), $C_2H_3^+$ (m/z = 27), $C_2H_4^+$ (m/z = 28) and $C_2H_5^+$ (m/z = 29). The peak at 29 m/z is also probably indicative of the presence of ethylene (C_2H_4) in the sample, (c) NO^+ (m/z = 30.006) and CH_3NH^+ peaks, (d) formaldehyde (CH_2OH^+, m/z = 31), O_2 (O_2^+, m/z = 32) and methanol ($CH_3OH_2^+$, m/z = 33) peaks

styrene trimethyl benzene/ethyl toluene, Fig.S7a,b.

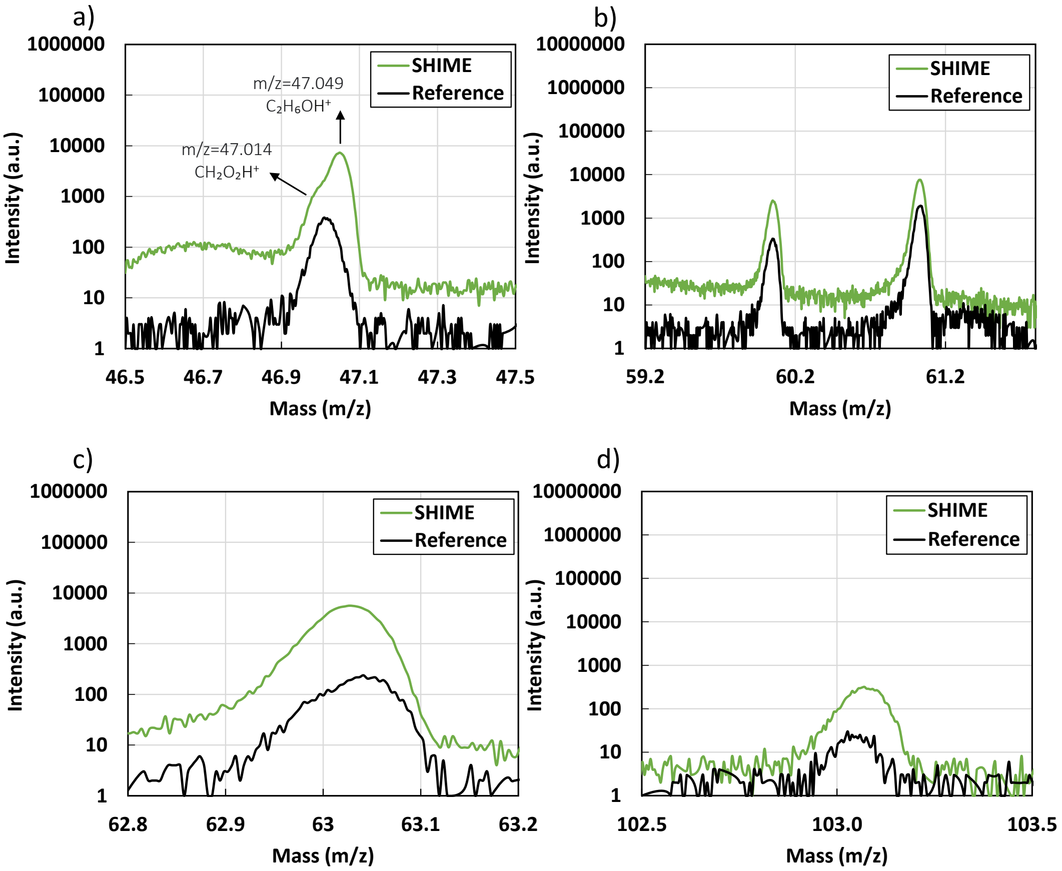

Figure S5. (a) formic acid $CH_2O_2H^+$ and ethanol $C_2H_6OH^+$ related peaks, (b) propylamine ($C_3H_9NH^+$, m/z = 60) and acetic acid ($C_2H_4O_2H^+$, m/z = 61) related peaks, (c) dimethyl sulfide ($C_2H_6SH^+$, m/z = 63) peak and (d) pentanoic acid ($C_5H_10O_2H^+$, m/z = 103.

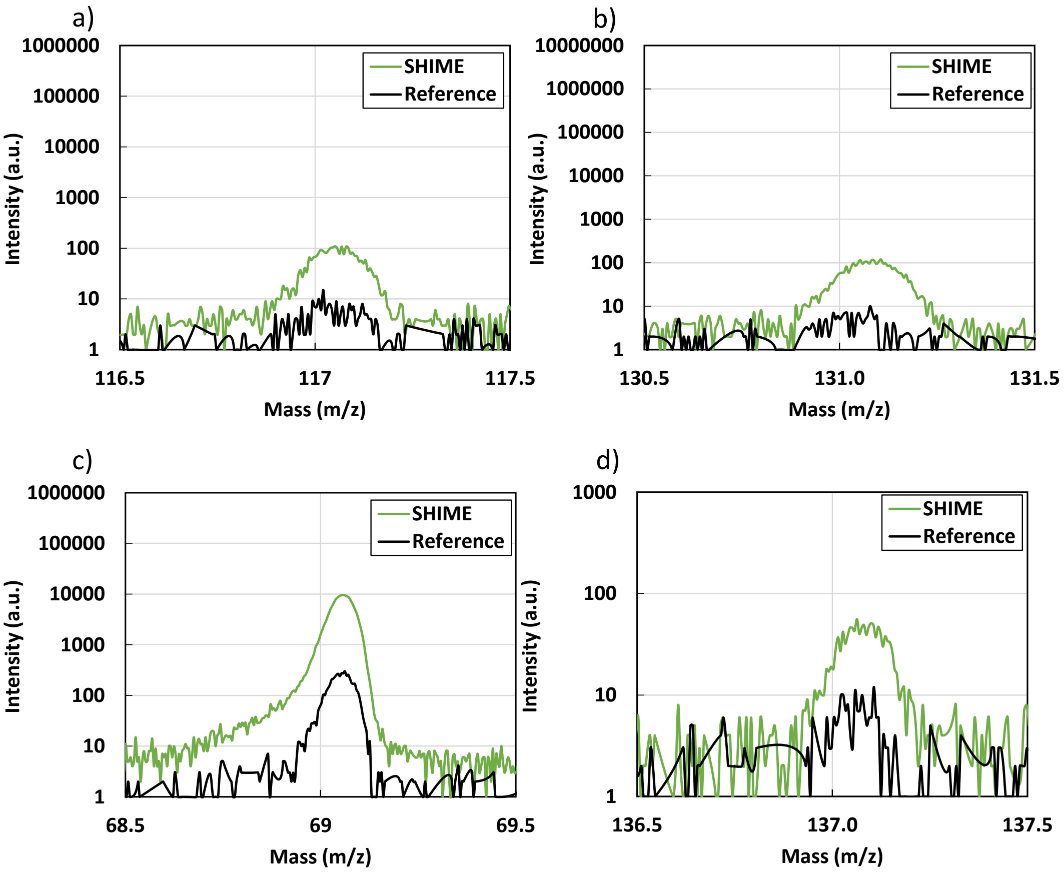

Figure S6. (a) hexanoic acid ($C_6H_12O_2H^+$, m/z = 117), (b) heptanoic acid ($C_7H_14O_2H^+$, m/z = 131), (c) isoprene ($C_5H_8H^+$, m/z = 69) and (d) pinene/limonene ($C_10H_16H^+$, m/z = 137) peaks

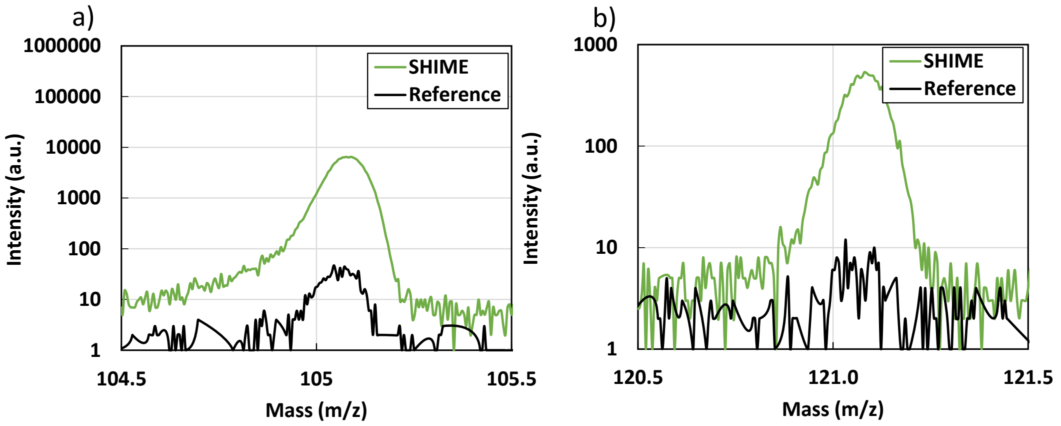

Figure S7. (a) styrene ($C_8H_8H^+$, m/z = 105) and (b) trimethyl benzene/ethyl toluene ($C_9H_12H^+$, m/z = 121) peaks
